# Supplementary material for: CellMet: Extracting 3D shape and topology metrics from confluent cells within tissues
Source: PLoS Comput Biol. 2025 Jul 30;21(7):e1013260. doi: 10.1371/journal.pcbi.1013260 (PMC12327599; doi:10.1371/journal.pcbi.1013260)
Supplement: S1 File — (PDF) [file pcbi.1013260.s001.pdf]

Supplementary Information

CellMet: Extracting 3D shape and topology metrics from confluent cells within tissues

Sophie Theis[1*], Mario A Mendieta-Serrano[1], Bernardo Chapa-y-Lazo[1], Juliet Chen[1,2], Timothy E Saunders[1*],

1 Warwick Medical School, University of Warwick, Coventry, United Kingdom **2** London Centre for Nanotechnology, Department of Cell and Developmental Biology, UCL, London, United Kingdom

Experimental Methods

Image acquisition

All images were visualised using an Olympus spinning disk confocal system with a confocal scanning W1 unit (Yokogawa), except the *Drosophila* mesoderm invagination described in [1]. Either 60x/1.30NA or 40x silicon immersion objectives were used, with a Hamamatsu ORCA-FusionBT C15440 Digital Camera.

Drosophila mesoderm invagination

The images on *Drosophila* mesoderm invagination are created from raw data shared with permission from the Suzanne lab [1].

Drosophila heart

A stage 16 *Drosophila* embryo resulting from the cross of lines TSD0019 (w*; UASp>CIBN-GFP; Sb/TM3,Ser) and TSD0009 (HandGal4/TM3 (III)) was dechorionated using 50% bleach, attached on the dorsal side to a 35 mm glass-bottom imaging dish (FluoroDish FD35, World Precision Instruments) using Heptane glue and covered with Halocarbon oil 700 (H8898, Sigma-Aldrich),

Zebrafish myotome

Whole wild-type zebrafish embryos were injected with *lyn-GFP* mRNA for labelling cell membranes. Injected embryos were incubated at 25°C until they were visualised. Embryos were mounted in low melting agarose and were bathed with embryo medium containing 0.003% tricaine.

hESCs on micropatterns

Micropatterned colonies were generated according to [2]. Briefly, plain coverslips were incubated with PLL(20)-g[3.5]-PEG(5) and placed on a UV-Ozone activated chrome mask, then exposed to UV for 8 minutes to generate micropatterned coverslips, which were then coated with 10% rh-Laminin-521 (v/v, Thermo Fisher Scientific). Human ESCs (H9 line - WiCell) were passaged using Accutase (Stemcell Technologies), diluted in StemFlex with 10 µM Y-27632, and adjusted to 600,000 cells/mL. After 3 hours, media was replaced with fresh StemFlex medium. 3 days later (∼70 hours), micropatterned hESC colonies were fixed in 4% paraformaldehyde and stained using anti-human beta-catenin antibody (R&D Systems) and secondary antibodies conjugated with Alexa Fluor 647.

3D segmentation with CellPose

Single cell segmentation was performed using the Cellpose 3.0 algorithm [3]. We generated custom models to apply to our data, starting from the *cyto3* model. For each tissue, we then performed manual correction using the Cellpose GUI. Final models were used for prediction of volumetric stacks creating 2D labels on each XY slice. These were then stitched together using the following parameters (diameter=30, flow threshold=0.4, cellprob threshold=0, stitch threshold=0.1). Labels were saved as multi-page tif files for analysis.

References

1. Mélanie Gracia, Sophie Theis, Amsha Proag, Guillaume Gay, Corinne Benassayag, and Magali Suzanne. Mechanical impact of epithelial- mesenchymal transition on epithelial morphogenesis in drosophila. *Nature Communications*, 10:2951, 2019.

2. Tiago Rito, Ashley RG Libby, Madeleine Demuth, Marie-Charlotte Domart, Jake Cornwall-Scoones, and James Briscoe. Timely tgfβ signalling inhibition induces notochord. *Nature*, pages 1–10, 2024.

3. Carsen Stringer, Tim Wang, Michalis Michaelos, and Marius Pachitariu. Cellpose: a generalist algorithm for cellular segmentation. *Nature Methods*, 18(1):100–106, 2021.
