# Supplementary figures and images for: CellMet: Extracting 3D shape and topology metrics from confluent cells within tissues
Source: PLoS Comput Biol. 2025 Jul 30;21(7):e1013260. doi: 10.1371/journal.pcbi.1013260 (PMC12327599; doi:10.1371/journal.pcbi.1013260)

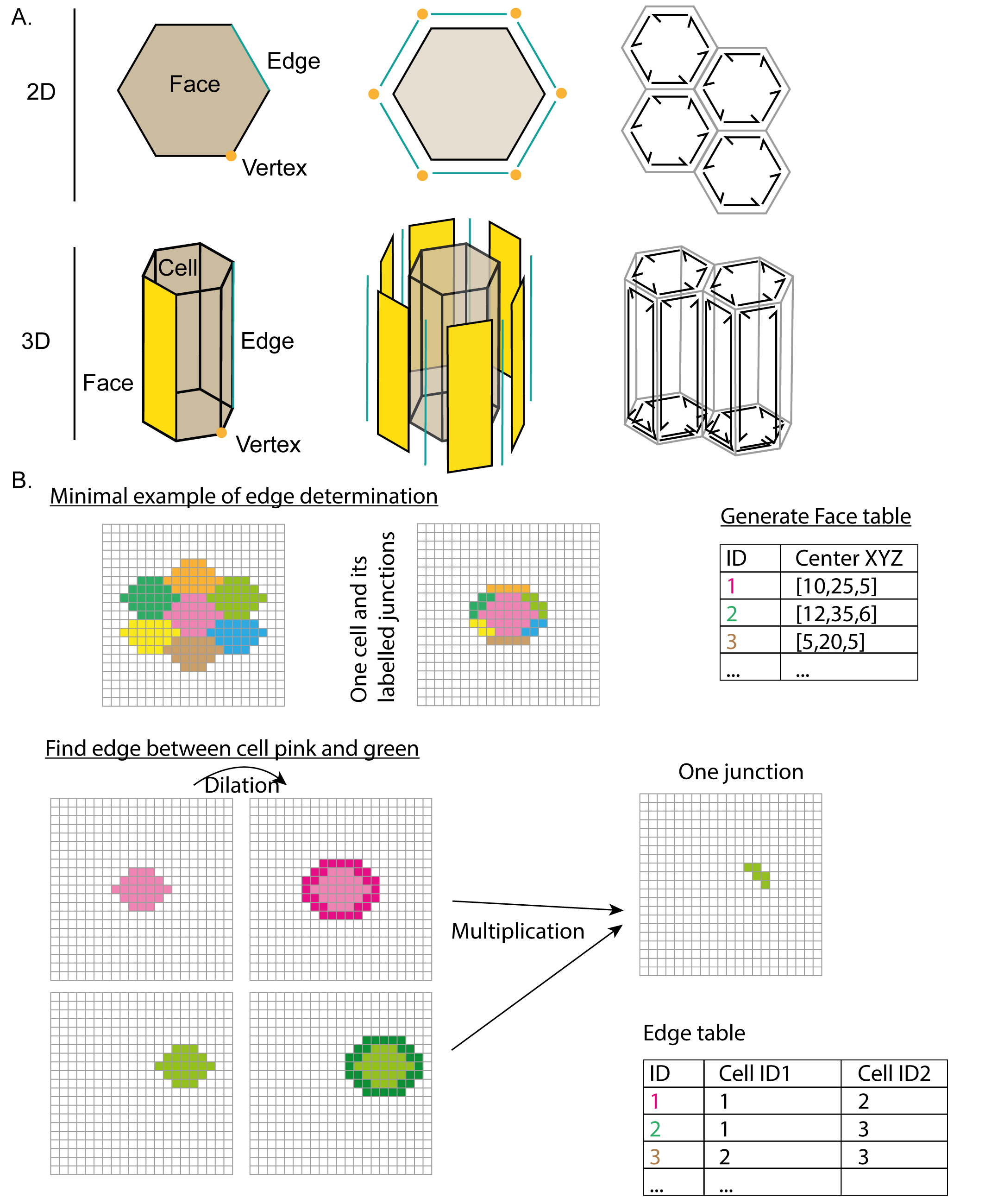

Supplement: S1 Fig — A. Cell decomposition into faces, edges and vertices in 2D (top) and 3D (bottom). B. Minimal example of how edge is determined in 2D using CellMet. (TIF) [file pcbi.1013260.s002.tif]

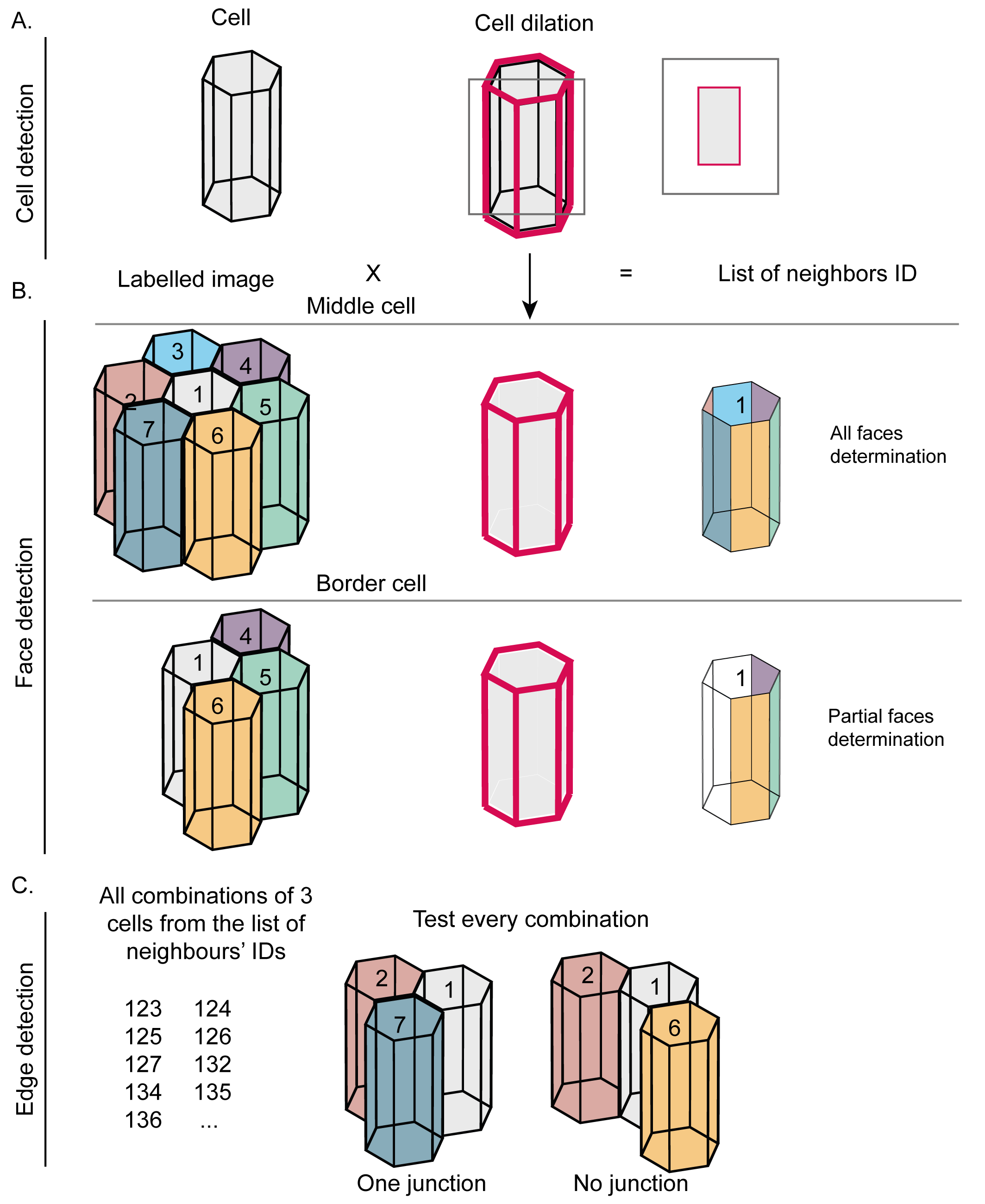

Supplement: S2 Fig — A. Cell detection is based on the segmentation. Cells dilated by one pixel are generated to facilitate face and edge detection. B. Faces are found by multiplying the labelled image by one dilated cell. Non-zero pixel values correspond to the neighbouring cell ID. Cells at the edges will not have all their faces detected (bottom). C. From the list of neighbouring cells, we find all combinations of 3 cells and multiply these 3 dilated cells. Pixels remain when they form a junction, otherwise they are removed to indicate empty space. (TIF) [file pcbi.1013260.s003.tif]

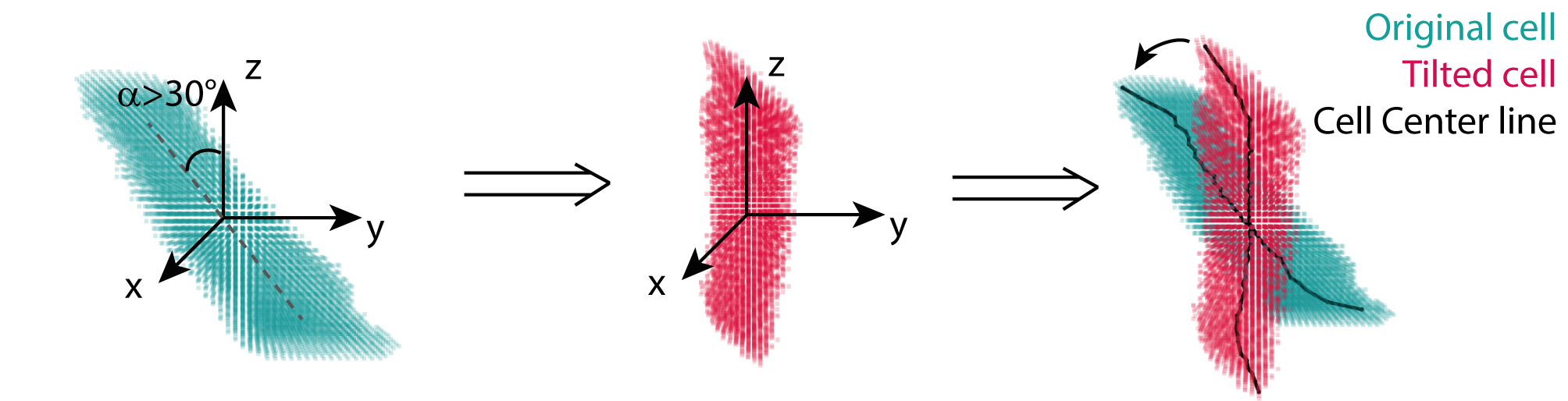

Supplement: S3 Fig — A tilted cell can lead to miscalculation of the cell centreline (left). If the major axis orientation is >30∘ to the z-axis, then CellMet reorients the cell (middle). The cell centreline is calculated and then put back to the original orientation (right). (TIF) [file pcbi.1013260.s004.tif]
