# Supplementary material for: CellMet: Extracting 3D shape and topology metrics from confluent cells within tissues
Source: PLoS Comput Biol. 2025 Jul 30;21(7):e1013260. doi: 10.1371/journal.pcbi.1013260 (PMC12327599; doi:10.1371/journal.pcbi.1013260)
Supplement: Attachment — Submitted filename: Response to Reviewers.pdf [file pcbi.1013260.s005.pdf]

Authors Response to Reviews

April 2, 2025

Dear Editors and Reviewers,

We thank the Reviewers for their detailed and constructive comments on our original submission. We have substantially reworked the manuscript to address the Reviewer concerns and to align with PLoS Computational Biology format. We believe the paper is now significantly improved and we hope suitable for PLoS Computational Biology.

Below, we provide a point-by-point response to the reviewers, with blue text corresponding to our responses. Major changes in the manuscript are highlighted in red.

Sincerely,

Sophie Theis and Timothy Saunders

Reviewer 1

This paper presents a python software library for analyzing cells in 3D. I think the paper needs major revisions for publication.

Major general problems.

- 1st. The structure itself does not fascilitate understanding what the package is doing.
- 2nd. It is not clear to me what measurements are being made and what approximations they use.
- 3rd. The end of the paper says that the software is for "general users" which is too vague.

As outlined below, we have made significant changes to the structure. This hopefully makes the metrics employed and the generality of our approach more apparent.

Improving the structure of the paper.

The authors reference a decent number of publications that use similar measurements and analysis. I know that these publications have made their software available too. Something that could help would be a table with types of measurements used for analyzing epithelia vs software packages.
Say which features each software package supports or doesn't support.
I think this would greatly improve the readability of the paper and show what is novel about it. What about cell profiler or CartoCell? They reference Scutoids, what about the analysis performed in that paper.
We thank the Reviewer for suggesting this. We have added the following table into the manuscript. It helps to see what our method brings compared to other approaches and the limitations, (lines 224-232).

	CellMet	CellProfiler	CartoCell	Griottes	iPS cells[1]
Volume	✓	✓	✓	✓	✓
SurfaceArea	✓	✓	✓	✓	
Aspect ratio	✓	2D only	✓		✓
Cell solidity	✓	2D only	✓		
Cell orientation	✓	2D only	✓	✓	✓
Cell eccentricity	✓	2D only		✓	
Fluorescence				✓	
Number of neighbours	✓	✓	✓		
Connectivity graph	✓			✓	
Edges (decomposition)	✓			✓(length)	
Faces (decomposition)	✓				

Table 1: Table of features measured by different software.

	CellMet	CellProfiler	CartoCell	Griottes	iPS cells[1]
2D		✓		✓	
3D	✓	✓	✓	✓	✓
Single Cell		✓			✓
Cell in epithelium	✓	✓	✓	✓	✓

Table 2: Kind of cells/tissue that we measure.

Clear description of what measurements are, how they're calculated and what approximations are being made.

I did not understand how does a general blob of pixels get transformed into a table of faces, edges and vertexes representation. Could this demonstrated with some very simple geometries? Eg. A sphere? Or if this is not applicable for a sphere, the authors should be clear about what it is applicable.

Determining faces, junctions and vertices is based on the multiplication of images and the pixels that remain. CellMet's underlying method is based on the neighbourhood of the cells, assuming they are tightly packed. We assign a unique ID to those pixels that are shared by multiple cells, which is stored in a lookup table along with information about the cell morphology and ID (see Reviewer Figure 1). CellMet is not designed for analysis of faces, edges and vertices of an isolated cell as it identifies the cell shape through the interaction with neighbouring cells. We have clarified these points on (lines 67-92).

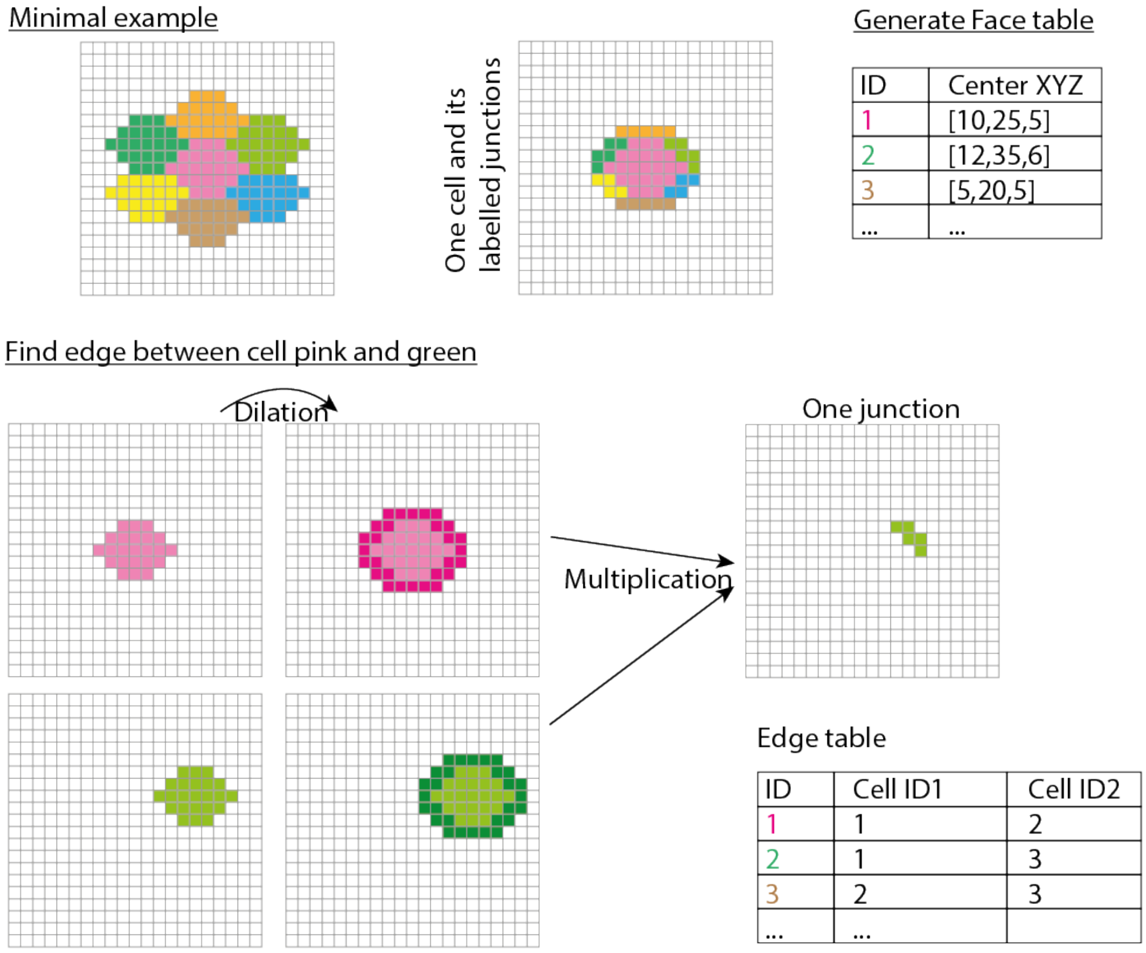

Figure 1: Minimal example of how we determine an edge in 2D using CellMet.

The description for generating surfaces seems to rely exclusively on the overlap of 1px dialated cell-cell overlap. I could see this causing some problems with finer features.
The Reviewer is correct that this version of CellMet relies on 1 pixel dilated overlap; it only works for tightly packed tissues. This approach works very well for a broad range of tissues across different organisms/systems, as we show in the manuscript. This method can be improved, for example by allowing flexibility in defining the cellular domain. This will add an additional parameter, defining the region around a cell to be explored, and the output will be tissue-dependent. Further, our current approach makes defining edges and faces straightforward; allowing the presence of extracellular space represents a significant challenge in the analysis and is more appropriate for a later version.

The Reviewer is correct that the structure of CellMet is not suitable for analysing tissue with finer features, such as filopodia. Software such as FiloDetect [2] are designed to analyse such specific structures. We have added a new paragraph in the main text to highlight limitations and constraints of our methods (lines 240-244).

The determination of edges looks at 3 clusters of cells. It seems like this isn't sufficient for 3D environments, for example scutoid cells.
In 3D, an edge is defined as the intersection of three (or more) cells. For scutoid cells, the edges are still defined by three cells. However, one edge will not follow the entire length of the cell in the case of an epithelial cell (see Reviewer Figure 2). In some cases, more than three cells are connected to the same edge, *e.g.* rosettes. These organisations are usually transitional [3] and so we do not explicitly account for them here. We have clarified these points on (lines 80-83).

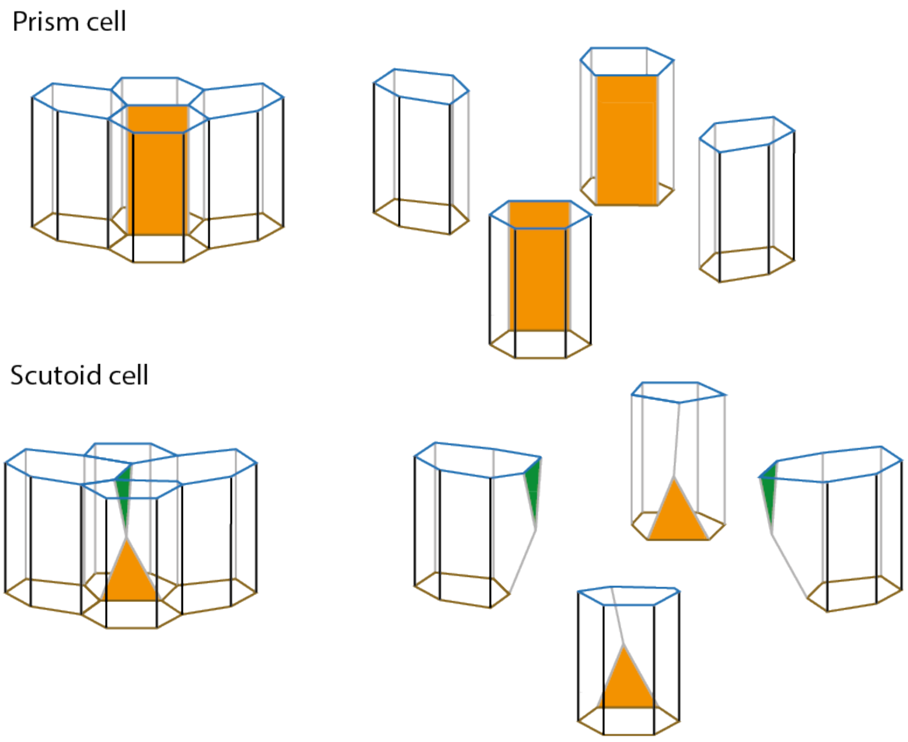

Figure 2: Illustration of cell organisation around edge for prism and scutoid cell.

"Our approach requires cells to be tightly packed;" Are there other requirements? There also is a statement about protrusions or processes. Do you have a specific metric that would let somebody eliminate this algorithm, or possibly refine their segmentations to be more suitable?

We have added a "Limitations and constraints" paragraph to let readers know if this method is right for them, see (lines 245-250). This also builds on the newly added Table of method comparison.

In figure 3, the connectivity has " The sharp increase is not observable by considering the apical surface" Maybe this could be illustrated with a cell that has neighbors that are not connected in an apical only analysis.

At this stage, all cells that we have segmented have a visible apical cell surface. It has been shown during mesoderm invagination, cells maintain their apical connections and they neither divide nor exchange their neighbor [4]. We extracted the apical cell surface of our segmentation and measured the number of neighbours. We did not observe any difference between the two stages (Reviewer Figure 3). This quantification has been added to Figure 3 in the main text to highlight this point.

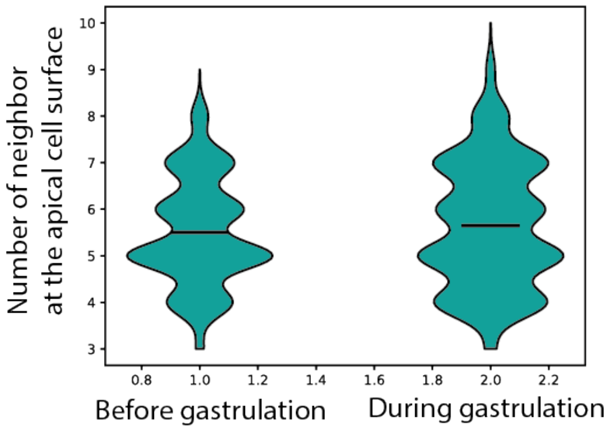

Figure 3: Measurement of cell neighbour number at the apical surface of the cell during gastrulation in *Drosophila*. $n \simeq 900$ cells from 1 embryo.

"Further, we calculated the connectivity graph (Fig 5A)" this is a good candidate to compare to other software packages that can create a connectivity graph. Does it exist? The only comparison is "This provides a much quicker method to assess cell alignment, compared to human-annotated approaches"

Connectivity (or adjacency) graphs are used during segmentation [5, 6, 7]. While not unique, CellMet offers a quick approach for generating such connectivity graphs. We have clarified these points on (lines 73-80).

In some tissues, such as the forming *Drosophila* embryonic heart, it is challenging to identify cell neighbours quickly, especially in the absence of specific cell fate markers. The connectivity graph produced by CellMet enables rapid insight into cell-cell contacts and highlight any misalignment, which is an important input for quantitative approaches [8]. We include discussion on (lines 219-230).

If this is going to be used as a general software package, it should explore the limitations in a more systematic and quantitative manner.

As suggested, we have added a paragraph to explore the limitation of our software (lines 240-256) along with the new table comparing different methods.

Who are general users?

By addressing the first part, and the second part, this part should come out naturally. Do the authors want to target biologists with segmented data who can use your package and compliment an existing work flow or repeat measurements from another publication? Is it more for a technical audience. Somebody who might use the software as a library or a peice of an existing workflow.

We wanted to target both audiences, which partly explains the lack of clarity in the original submission. We wanted to develop an easy-to-use tool for biologists to extract useful information from their segmented data. We also know that people may want to measure parameters that we have not anticipated. Therefore, the software is written in an open manner, enabling those with more technical experience to adapt and improve the software to suit their needs. We emphasise these different points of focus in the Introduction, Lines 45-46.

Conclusion

I cannot really decide who the target audience of this paper would be. Do they have a really strong algorithm they want to publish? It isn't highlighted. Do they have a software tool that somebody should be using to reproduce some specific measurements? That is where a table could help.

We hope that with the changes outlined above (and also related to Reviewer 2), we have addressed this concern. To be clear, our primary goal was to generate a software package that would enable biologists to maximise the information they can extract from 3D segmentation of dense tissues. We know from engagement with the community before and since releasing CellMet that there is significant demand for such a tool. In addition, it is important that CellMet is open and accessible for personalisation to particular researcher needs.

Reviewer 2

The authors present "CellMet", a software written in Python for the analysis of the shape of cells and tissues based on 3D images. The introduction presents the context and the need for quantification of 3D shape. The different sections present the methods used for obtaining the 3D images used within the manuscript, a description of the software design and of the features that can be obtained. An applications sections describes a variety of use cases for the software.

The main originality of the proposed software seemd to be the quantification of the relationships between cells within 3D images. While this question is of interest for a variety of applications, the manuscript does not appear to be acceptable for publication in its present form, for several reasons:

Comments

The title mentions cell shape, and the introduction presents cell morphology. However most of the proposed metrics concern the arrangement of cells and the topology of the tissue. This could be more clearly emphasised
Cell morphology and topology of the tissue are closely linked, as tissue topology (*connectivity*) depends on cell shape. Further, our methodology is focused on cell morphology within a tissue. This is partly why we chose CellMet, as a shortened form of Cell Metrics, as our measures encompass both geometric and topological quantities. CellMet is not designed to analyse isolated cells, especially as tools have already been developed for this type of data [9] (see also response to Reviewer 1). Instead, CellMet is focused on extracting quantitative data about cells within tightly packed tissues. As part of this, we include cell shape metrics, such as elongation and twist. We agree with the Reviewer that the original title was not sufficiently clear, and we have updated the title.

The structure of the manuscript makes it difficult to follow. It is surprising that after an introduction presenting the software as main subject, the next section are methods presenting preparation of images. Explaining the meaning of the images in the introduction would clarify.
The structure of the manuscript does not follow the recommendation of the journal for "Software" article. Using the recommendation could be an option to clarify the structure.
We have updated the organisation of the manuscript to follow the recommendation of the journal for a "Software" article. We keep the "Methods" section but it now only includes information related to the experiments and it has been moved to the end of the manuscript. As this tool is aimed primarily at end-users, we feel it is important to have a range of applications shown.

The authors use volume and surface area as base metrics, and mention spherical harmonics. It is however very surprising that they do not mention other "classical" features used for morphology analysis of 3D regions. Examples are equivalent ellipsoid, thickness, Feret diameters... While they are not the main topic of the proposed software, they should be mentioned if using "shape metrics" in the title.
We have updated Figure 2 to present all features currently included in CellMet. Though this list is extensive, it is not exhaustive. We are keen to support requests sent through the Github page, so we can add new metrics as needed; this is clarified on Lines 265-267. Further, the open nature of CellMet means that experienced users can add additional metrics if required. Lastly, we utilise the general csv file format, enabling new measures to be completed outside of CellMet.

The authors seem to be totally unaware of the notion of "Region Adjacency Graph", which is a very classical approach in image processing, and that appears to be the main data structure of the proposed software.
Region adjacency graph (RAG) and connectivity graph (CG) refer to similar theory/principles. RAG represents the adjacency relationships between different segmented regions while CG represents the connectivity of individual pixels/voxels based on predefined neighbourhood rules. For example, the segmentation tool Ilastik uses RAG to agglomerate labels based on edge features. We agree that we

should have referenced RAG explicitly rather than just CG. We note that these terms are used in similar approaches: RAG [5, 10]; CG [11, 12, 7]. We have added a paragraph in the main text to explain the construction and the use of our connectivity graph (Lines 73-80).

Finally, in some cases the authors use methods that may provide strong bias, with the aim of modelling biological processes. This approach can lead to erroneous conclusions, and the authors must be aware of the possible errors that can be provided by the image analysis methods they use.
We agree that there are potential issues, and we now highlight these more clearly (see response to Reviewer 1). Here, we have focused on improving the ease with which useful information about cell shape metrics within dense tissues can be extracted. This is a very important yet difficult step, especially in analysing 3D segmented data. Of course, as with nearly all attempts to quantify biological processes, we need to make assumptions and approximations.

Other comments

abstract: it could be mentioned in the abstract that the proposed software is written in python
This is a very good point and we have added to the abstract.

L144+: it is not very clear how the transition between the 3D label map and the topological data structure is made. The process of identifying voxel "types" (i.e. within cell, face, edge or vertex) is clear. However, the process of linking the edges together to consider a curve does not seem to be explained. Otherwise, I do not understand how the "length" of the edge is computed.
Each edge is defined between two vertices. We calculated two lengths for each edge: (1) the shortest straight line distance between the two vertices; and (2) the real edge length, following any curvature of the edge. We calculated the "real" length of the edge by computing the Euclidian distance point by point. We are aware that this measure may be an overestimate; to limit this, the curve is smoothed. We apologise that the methodology for defining edge lengths was not clear. "The edge table is used to link cell and face tables" refers to the methods used to store the data. The half-edge vertex model or edge vertex model, is a data structure that maintain incidence information of edges, faces and cells. Edges are the link between faces and cells. This structure ensures quick and efficiency access to data. It is motivated by mapping onto vertex-models of tissues. We have clarified this in the revised manuscript, Lines 67-71.

Also, I strongly suggest dissociating the notion of "half-edge", which a specific implementation, from the connectivity graph. The authors may also consider the notion of "cell complex", that is more formally defined, and that can be considered as a generalisation of polygon meshes.
The information in the connectivity graph and what is in our half edge structure are different. Our connectivity graph corresponds to what the reviewer also called the Region Adjacency Graph. In both cases, $G(V, E)$ are graphs, where vertex $v_i \in V$ represents the i-th cell and edge $e_i \in E$ connects vertex together. On the other hand, the half-edge structure is used to describe the actual geometry of cells. It could be described as $G(C, F, E)$, where cell $c_i \in C$ represents the i-th, e_i and f_i the edge and face of a cell. In Reviewer Figure 4, we highlight the differences between the two representations. Of course, the two representations are closely related and can be mapped onto each other. The half-edge structure can be turned into polygon meshes, in order to be exported and used in other software packages for visualisation (*e.g.* using the Python library *meshio*).

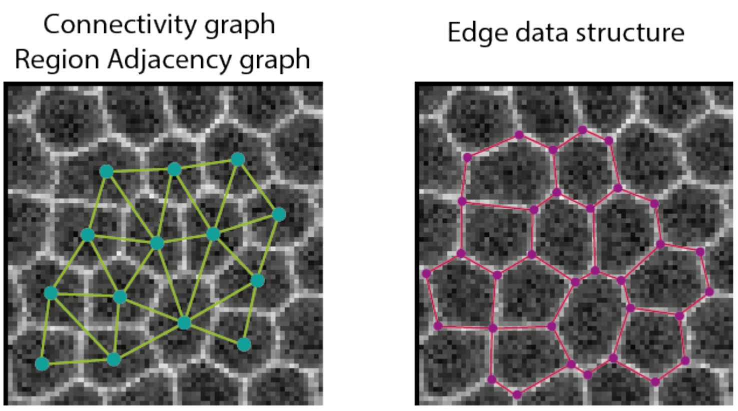

Figure 4: Comparison of Connectivity graph and edge structure.

L155: this is a classical method for building region adjacency graph. At least the term should be mentioned. Reference papers can also be cited.
We agree with the Reviewer, and we now include references in the revised manuscript.

L173: I do not understand the meaning of the last two sentences.
Our method relies on cells being in contact to determine a contact surface (face) or an intersection between three cells (edge). So, for a cell at the edge of the segmentation, CellMet will not be able to determine faces and edges where there are no neighbouring cells. We have added an explanatory case in Figure S2B and rephrased in the main text, lines 67-92. We aim to introduce boundary constraints in the next iteration of CellMet, but that represents a substantial piece of work in its own right.

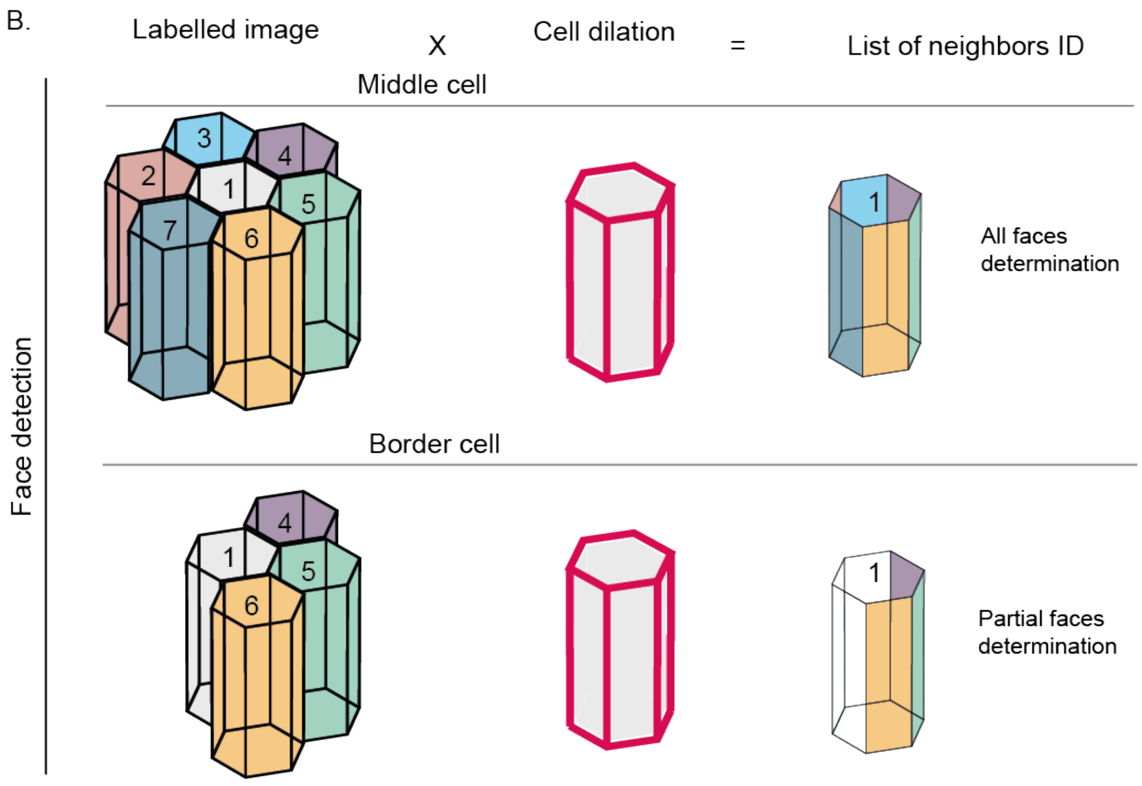

Figure 5: Example of face detection for cell in middle of tissue (top) and for a cell at a boundary, with neighbours only on one side (bottom).

L178. This paragraph is surprisingly very short regarding the manuscript title. Authors should be aware that it is not necessary to resize/resample the image to measure the volume.

We have now expanded on this section (lines 94-128), highlighting that such measurements can be done independent from the scale. As we resample to measure some metrics, we calculate the volume similarly.

L183: evaluating the surface area by simply counting the number of boundary voxels leads to a strong overestimation. If the purpose is simple to compare populations of cells, this is fine. But as the authors aim to link with modelling approaches, this bias should be taken into account and commented. Check for example Joakim Lindblad & Ingela Nyström "Surface area estimation of digitized 3D objects using local computations", 2002, or methods based on discretization of Crofton formula, such as C. Lang, J. Ohser, R. Hilfer, "On the analysis of spatial binary images", Journal of Microscopy, vol 203, 2001.

We thank the Reviewer for highlighting this and providing specific references. We agree that our approach will tend to overestimate, as we include all potential voxels. We have added a comment about the overestimation of this measure, lines 98-99.

L184: using the maximum value of the distance transform does not yields the centroid, but the center of the largest inscribed ball or sphere.

We thank the Reviewer for highlighting this error. As we consider cells within tightly packed confluent tissues, our measure is close to the cell centroid. Motivated by this comment, we now include in CellMet the cell centroid, inferred from the centre of mass of the cell volume.

Further, we noticed that using the EDT method tended to an overestimation of the real length of the cell, while the shortest distance is similar ($\pm 0.5m$)(Reviewer Figure 6). We have updated our method: we now calculate the centre of each plane to create the line through the middle of the cell ("mean" in Reviewer Figure 6). We also better account for length estimates for cells that are orientated away from the z-axis through rotation transformations ("Rot" in Reviewer Figure 6). Finally, we have added a smoothing filter before calculating any length in order to reduce length over-estimation. Full details are provided in lines 95-105.

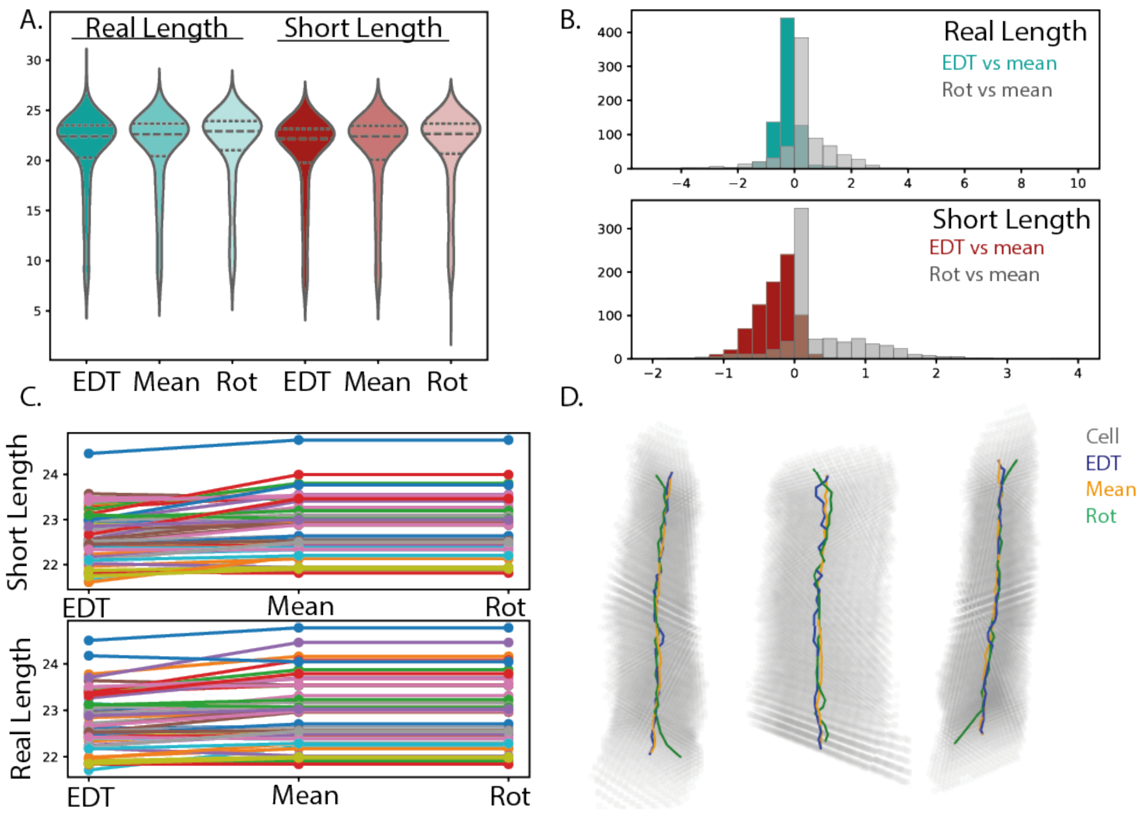

Figure 6: Comparison of EDT, mean and rotation methods in *Drosophila* during mesoderm invagination. A. Violin plot of the length measurement for different method. B. Histogram of the difference between EDT/Rot and mean methods for both length measurement. C. Examples of values measured with the different methods. Dots are linked together to show the difference. D. Example of the different center line obtain with the three methods.

L187: it is not very clear what the author want to quantify within this sentence.
This part of the paragraph has been modified to include the notion of the Centerline.

L189: computing an equivalent ellipsoid would provide a 3D orientation, not a series of orientations along various planes, which is less generic.
In order to provide a more generic 3D orientation, we provide in addition rotation in the spherical coordinates system ρ, θ, φ, where ρ is the radial distance, θ is the polar angle, and φ is the azimuthal angle.

L190: (more a question). Do the author refer to a specific definition for the sphericity? Common software such as ImageJ use definition for (2D) circularity as $4 * pi * A/P^2$. I would have expected a straightforward extension to 3D, but I am not sure a consensual definition exist.
We used the sphericity defined by Wadell : $\frac{\pi^{1/3} \times 6V^{2/3}}{A}$, where V and A are the volume and the surface area of the cell respectively[13]. We agree with the Reviewer that there is not a clear consensual definition in 3D. For example, MorphoLibJ use an other definition : $36\pi\frac{V^2}{A^3}$. We clarify this on lines 112-114.

Fig. 2: the notion of "edge curvature" is IMHO erroneous. The curvature is defined locally, for each point of the curve (or of a surface). I would rather speak of "tortuosity", or maybe another term ("straightness"?)
We thanks the reviewer for pointing out this error. We change the term "edge curvature" for "straightness".

Fig.4: the authors propose a very specific shape analysis to distinguish between scutoid and prism shapes. I would have appreciated a comparison with more classical shape

features.

We chose prism and scutoid shapes as there are shapes commonly found in epithelium [14, 2, 15]. We have updated Figure 4 to include more shape features to compare these two categories of cell shape.

It would be fine to compare the software with other software that allow management and analysis of topological arrangement of cells. See e.g. "Image Processing Filters for Grids of Cells Analogous to Filters Processing Grids of Pixels", by Robert Haase, in Frontiers in Computer Sciences (2021), or "Griottes: a generalist tool for network generation from segmented tissue images", by Gustave Ronteix in BMC Biology (2022).

This comment is similar to Reveiwer 1. We have added a comparison table to the manuscript to compare the software.

References

[1] Matheus P Viana, Jianxu Chen, Theo A Knijnenburg, Ritvik Vasan, Calysta Yan, Joy E Arakaki, Matte Bailey, Ben Berry, Antoine Borensztejn, Eva M Brown, et al. Integrated intracellular organization and its variations in human ips cells. *Nature*, 613:345–354, 2023.

[2] Sharmin Nilufar, Anne A. Morrow, Jonathan M. Lee, and Theodore J. Perkins. FiloDetect: automatic detection of filopodia from fluorescence microscopy images. 7(1):66.

[3] Molly J. Harding, Hillary F. McGraw, and Alex Nechiporuk. The roles and regulation of multicellular rosette structures during morphogenesis. 141(13):2549–2558.

[4] Matteo Rauzi, Ana Hočevar Brezavšček, Primož Ziherl, and Maria Leptin. Physical models of mesoderm invagination in drosophila embryo. 105(1):3–10. Publisher: Cell Press.

[5] Jiaxiang Jiang, Amil Khan, S. Shailja, Samuel A. Belteton, Michael Goebel, Daniel B. Szymanski, and B. S. Manjunath. Segmentation, tracking, and sub-cellular feature extraction in 3D time-lapse images. *Scientific Reports*, 13(1):3483, March 2023. Publisher: Nature Publishing Group.

[6] Katarzyna Hajdowska, Sebastian Student, and Damian Borys. Graph based method for cell segmentation and detection in live-cell fluorescence microscope imaging. *Biomedical Signal Processing and Control*, 71:103071, January 2022.

[7] Rodrigo Cilla, Vinodh Mechery, Beatriz Hernandez de Madrid, Steven Del Signore, Ivan Dotu, and Victor Hatini. Segmentation and Tracking of Adherens Junctions in 3D for the Analysis of Epithelial Tissue Morphogenesis. *PLOS Computational Biology*, 11(4):e1004124, April 2015. Publisher: Public Library of Science.

[8] Sham Tlili, Murat Shagirov, Shaobo Zhang, and Timothy E Saunders. Interfacial energy constraints are sufficient to align cells over large distances. *Biophysical Journal*, 2025.

[9] Matt De Vries, Lucas Dent, Nathan Curry, Leo Rowe-Brown, Vicky Bousgouni, Adam Tyson, Christopher Dunsby, and Chris Bakal. 3d single-cell shape analysis using geometric deep learning. *bioRxiv*, 2023.

[10] Adrian Wolny, Lorenzo Cerrone, Athul Vijayan, Rachele Tofanelli, Amaya Vilches Barro, Marion Louveaux, Christian Wenzl, Sören Strauss, David Wilson-Sánchez, Rena Lymbouridou, Susanne S. Steigleder, Constantin Pape, Alberto Bailoni, Salva Duran-Nebreda, George Bassel, Jan U. Lohmann, Miltos Tsiantis, Fred A. Hamprecht, Kay Schneitz, Alexis Maizel, and Anna Kreshuk. Accurate and versatile 3D segmentation of plant tissues at cellular resolution. *eLife*, 9:1–34, July 2020.

[11] Meng Lu, Charles N. Christensen, Jana M. Weber, Tasuku Konno, Nino F. Läubli, Katharina M. Scherer, Edward Avezov, Pietro Lio, Alexei A. Lapkin, Gabriele S. Kaminski Schierle, and Clemens F. Kaminski. ERnet: a tool for the semantic segmentation and quantitative analysis of endoplasmic reticulum topology. *Nature Methods*, 20(4):569–579, April 2023.

[12] Sören Strauss, Adam Runions, Brendan Lane, Dennis Eschweiler, Namrata Bajpai, Nicola Trozzi, Anne-Lise Routier-Kierzkowska, Saiko Yoshida, Sylvia Rodrigues da Silveira, Athul Vijayan, Rachele Tofanelli, Mateusz Majda, Emillie Echevin, Constance Le Gloanec, Hana Bertrand-Rakusova, Milad Adibi, Kay Schneitz, George W Bassel, Daniel Kierzkowski, Johannes Stegmaier, Miltos Tsiantis, and Richard S Smith. Using positional information to provide context for biological image analysis with MorphoGraphX 2.0. *eLife*, 11:e72601, May 2022.

[13] Hakon Wadell. Volume, shape, and roundness of quartz particles. 43(3):250–280. Publisher: The University of Chicago Press.

[14] Pedro Gómez-Gálvez, Pablo Vicente-Munuera, Antonio Tagua, Cristina Forja, Ana M. Castro, Marta Letrán, Andrea Valencia-Expósito, Clara Grima, Marina Bermúdez-Gallardo, Óscar Serrano-Pérez-Higueras, Florencia Cavodeassi, Sol Sotillos, María D. Martín-Bermudo, Alberto Márquez, Javier Buceta, and Luis M. Escudero. Scutoids are a geometrical solution to three-dimensional packing of epithelia. *Nature Communications*, 9:2960, July 2018.

[15] Pedro Gómez-Gálvez, Pablo Vicente-Munuera, Samira Anbari, Antonio Tagua, Carmen Gordillo-Vázquez, Jesús A. Andrés-San Román, Daniel Franco-Barranco, Ana M. Palacios, Antonio Velasco, Carlos Capitán-Agudo, Clara Grima, Valentina Annese, Ignacio Arganda-Carreras, Rafael Robles, Alberto Márquez, Javier Buceta, and Luis M. Escudero. A quantitative biophysical principle to explain the 3d cellular connectivity in curved epithelia. 13(8):631–643.e8. Publisher: Elsevier.
