# Supplementary material for: CellMet: Extracting 3D shape and topology metrics from confluent cells within tissues
Source: PLoS Comput Biol. 2025 Jul 30;21(7):e1013260. doi: 10.1371/journal.pcbi.1013260 (PMC12327599; doi:10.1371/journal.pcbi.1013260)
Supplement: Attachment — Submitted filename: Response_to_Reviewers_auresp_2.pdf [file pcbi.1013260.s006.pdf]

Authors Response to Reviews

June 12, 2025

Dear Editors and Reviewers,

We thank the Reviewers for their constructive comments on our resubmission to help the manuscript be clear and impactful. We have further reworked the manuscript to address the Reviewer concerns. We hope that this version is now suitable for PLoS Computational Biology.

We provide a point-by-point response to the reviewers, with blue text corresponding to our responses. Major changes in the manuscript are highlighted in red.

Sincerely,

Sophie Theis and Timothy Saunders

Reviewer 1

I feel the authors have adequately addressed my concerns. They do a better job of explaining the purpose of their software. I still feel there are still some issues concerning readablilty. Here are two examples:

Lines 111 to 119. The descriptions of the intermediate outputs (npz, obj, ply) seems to be largely irrelevant at this point. In contrast the statement "As CellMet does not correct for mis-segmentation; this need to be done prior to analysis." to be very important. I think this should be expounded upon, what are mis-segmentations? Each cell needs a unique label, the the labels need to be tightly packed, the epithelia needs to have a apical basil axis along the z-axis. Naively I take an image, I run it through cellpose and get a set of labels. Then I run it through CellMet is that going to work?

A mis-segmentation corresponds to missing cells (false negative) or cells segmented at the wrong position (false positive). The most recent segmentation methods (e.g., CellPose3) reduce the frequencies of such errors, but there are always still a few. Correcting these errors is time consuming as it requires manual correction. There are software packages, such as Napari, that can correct for mis-segmentation. CellMet is aimed at extracting quantifying the segmented data, not on the segmentation itself. We have moved the descriptions of the intermediate outputs in *Design and Implementation*. See (lines 69-73 and 79-86) in the revised manuscript.

Some of the 2D/3D confusion has statements like, lines 86-90. Is it useful for 2D analysis? It doesn't sound like it, but the authors don't want to let it go. Do the authors have code examples and jupyter notebooks that demonstrate 2D analysis? If they do some 2D examples there, then it might be worth including it in a section of it's own. "CellMet performs limited 2D analysis, xyz, found in our examples. Otherwise lines 86-90 should be reduced to "CellMet is not suitable for 2D analysis." Why say "it can do it"? It only weakens the other claims.

To clarify, we include 2D and 3D analysis tools as users will not always need to extract full 3D information from their segmentations. To avoid confusion, we have simplified the sentence at the beginning of the Design and Implementation section. We have also updated the sentence at the end of the cell straightness and sphericity section to distinguish more clearly the 2D/3D analysis. See (lines 63-68

and 150-153) in the revised manuscript.

The following details, I am a bit confused about, but the other reviewer seemed to be more expert on the topic.

Line 166, I don't understand the point of rotating. I can only guess that it is used in another calculation somewhere, but I don't understand why.
This rotation is made to limit error when calculating the centerline of the cell. Of course, this can be done in generalised coordinates, but we found it simpler to rotate into a specific axis.

Line 192 Was a PCA analysis performed? They also mention fitting the plane to the points and projecting the points onto the plane, is this part of the PCA analysis? Would it be possible to write the equation?
The projection onto the plane is not part of the PCA analysis. This is performed by a singular value decomposition (SVD) $A = USV^T$, where U, S and V are three matrices that transform the two orthogonal unit vectors to approximate the data sets. We use the SVD method to rotate data into the xy plane, and we remove the z-coordinates to project it. PCA analysis is used to measure how flat a face is; it is independent of the projection. We have added further details for the projection in the text along with references. See (lines 155-163) in the revised manuscript.

Reviewer 2

First of all, I would like to thanks the authors for the integration of the different remarks, that make the manuscript much easier to understand. However, there are still many parts that are difficult to understand, or that are too technical for the target audience. My main comment is that authors focus too much on the technical details, making it difficult for the reader to decide if the software may or not correspond to its needs.

We thank the Reviewer for the careful reading of the manuscript and helping us to maximise the accessibility. We have made the changes as suggested below.

L29-35. This paragraph is very important for understanding the manuscript, and should be emphasised. In particular, the lack of (user-friendly) software solution for analysing spatial arrangement of cells can be better discussed. We can find some tools for building region adjacency graphs, for examples, but this requires to build a complete workflow using scripting and/or programming. Also we can find some work related to the description of cell arrangement within tissues (see e.g. "Automatic identification and characterization of radial files in light microscopy images of wood", https://doi.org/10.1093/aob/mcu119, or "DRACO-STEM: An Automatic Tool to Generate High-Quality 3D Meshes of Shoot Apical Meristem Tissue at Cell Resolution, https://doi.org/10.3389/fpls.2017.00353). The notions of edges and faces should be introduced later, in the methods, as they describe a specific point of view for analysing the data.
We have rewritten this paragraph to highlight the gap in the area that our programme fills. We have included additional references to highlight current tools and how our programme addresses an important challenge (lines 34-49). The notion of edges and faces is now introduced later, at the beginning of *Design and Implementation* (lines 63-68).

L67: this paragraph is still difficult to understand, and needs rewriting for clarification. I suggest starting by explaining what features the authors want to quantify, and explaining how they are quantified in a second time. Here, the morphology of cells is described through that of the faces and edges they share with their neighbors. This requires to 1) identify neighbors, 2) use an appropriate data structure to represent how cells, faces and edges are related. The notion of half-edge is very technical, and should be introduced later. If it is used to describe edge/face morphology of individual cells, using "polygon

mesh" terminology makes the things much easier to understand.
We have rewritten the beginning of the Decomposing cell, edge and face section to clarity our point. We have separated the different concept to avoid confusion (lines 89-106).

L70: note that the topology is independent of the geometry, so writing that topology data contain shape information is a kind of non-sense...
This was a misuse of the term topology. We have rewritten this section.

L82: could be useful to explain / define what are prism or scutoid shapes earlier in the manuscript. As the scutoid seem to be a cell type specific to epithelia tissue, it could be beneficial to have a short explanation on such tissues (and the typical properties of epithelium cells) within the introduction.
We add a definition of prism and scutoid cell in the introduction, as well as a description of the conditions under which these structures can be found (lines 22-28).

a side remark on the violin plots of response documents: the plots depict distribution of integer values. Using violin plot is not the most appropriate for the nature of the data, due to the discrete nature of integers. A box plot would be better. In particular, as the smoothing factor used for box was chosen too small, this induces waviness in the representation that are particularly intriguing, and distract the reader from the main message.
We thank the reviewer for highlighting this. We agree about changing to improve presentation, and in Figure 3 we have changed the violin plot for a box plot.

L225-226: this sentence is an easy to understand summary of the different features the software provides. I strongly suggest to use it earlier, either in the introduction (typically around L39-40), or at the beginning of the "design and implementation" section.
A revised version of this sentence has been added at the beginning of Design and Implementation section (lines 63-66).

References
